# Multi-objective optimisation of reliable product-plant network configuration

**DOI:** 10.1007/s41109-017-0058-8

**Published:** 2018-01-15

**Authors:** Alexandra Brintrup, Alena Puchkova

**Affiliations:** Department of Engineering, Institute for Manufacturing, University of Cambridge, Charles Babbage Road, Cambridge, CB3 0FS UK

## Abstract

Ensuring manufacturing reliability is key to satisfying product orders when production plants are subject to disruptions. Reliability of a supply network is closely related to the redundancy of products as production in disrupted plants can be replaced by alternative plants. However the benefits of incorporating redundancy must be balanced against the costs of doing so. Models in literature are highly case specific and do not consider complex network structures and redundant distributions of products over suppliers, that are evident in empirical literature. In this paper we first develop a simple generic measure for evaluating the reliability of a network of plants in a given product-plant configuration. Second, we frame the problem as a multi-objective evolutionary optimisation model to show that such a measure can be used to optimise the cost-reliability trade off. The model has been applied to a producer’s automotive light and lamp production network using three popular genetic algorithms designed for multi-objective problems, namely, NSGA2, SPEA2 and PAES. Using the model in conjunction with genetic algorithms we were able to find trade off solutions successfully. NSGA2 has achieved the best results in terms of Pareto front spread. Algorithms differed considerably in their performance, meaning that the choice of algorithm has significant impact in the resulting search space exploration.

## Background

One of the major challenges in production planning is delivering orders to customers reliably whilst also minimising the costs involved in setting up and running the network of production across multiple-plants (e.g. Jordan and Graves 1995, Azaron et al. 2008, Lin et al. 2011).

Reliability of a delivery could be affected not only by one-off catastrophic incidents such as natural disasters or sociopolitical events, but much more frequent are everyday disruptions, such as resource breakdowns, worker absence, unstable manufacturing processes, shifting bottlenecks due to rush orders, product quality, IT system issues and so on, all resulting in lateness or lower quantity than what has been ordered. In the context of this paper we define reliability as the probability of an incident associated with inbound supplier failures resulting in the inability of the manufacturer to meet customer demand satisfactorily (Bundschuh et al. 2003, Zsidisin and Ellram 2003).

Reliability is the subject of several strands of diverse research in manufacturing engineering. Sourcing flexibility is concerned with distributing demand across multiple suppliers (Berger et al., 2004a, b), facility location models optimise the location of inventory taking into account demand and supply uncertainties (Snyder et al. 2006), vector assignment models distribute demand across locations (Pirkul 1989) and coverage models maximise the coverage of variable demand from a given set of locations (Daskin 1988). Process flexibility examines how capacity can match demand through redundant capability across locations (Jordan and Graves 1995). A neglected ingredient in these studies has been the distribution of multiple products across multiple suppliers to which a focal manufacturer must access for assembly.

For example in the automobile industry assignments of products to assembly plants show some degree of redundancy. Most plants build more than one product type and some products are built in more than one plant (Jordan and Graves 1995, Brintrup et al. 2016). Few studies that considered this bi-partite nature of the supply network modelled them as separately, whereas in reality both can happen at the same time (Masih-Tehrani 2011). Complex network topologies have not been taken into account, and studies mostly focussed on dyadic systems or multi-echelon chains (Stevenson and Spring 2007). Although cost minimisation is commonly deployed during the configuration process, reliability of production is rarely evaluated. One reason behind this is a lack of simple methods for incorporating reliability into the decision. Is the configuration of a given plant-product network reliable? Does the level of reliability justify costs involved in setting the network up and operating it? In this paper we attempt to provide a simple measure for assessing the overall reliability of a given network configuration, and a method to balance reliability and associated costs. The measure uses (i) individual supplier reliability scores determined a priori, (ii) information on possible products that plants can produce, and (iii) associated costs; to determine the overall reliability and cost of possible alternative network configurations. Using the measure, we then formulate the balancing problem as an optimisation problem and lay out the constraints, objective functions and variables that need to be solved. Three multi-objective genetic algorithms are compared in a case study from the automotive industry.

We have deliberately followed a minimalist approach and not taken into account fine-grained production parameters such as capacity, buffers and production rate, as our aim is to provide as generic a method as possible for use as a base model in case specific extensions. The paper is organized as follows: Section 2 positions our work in the context of reliable supply network design, and seeks analogies in network science studies, finding that methods from network science, while applicable, need careful thought before being transferred to product-plant network configuration problems because of the bipartite nature of the network. Section 3 then formulates the measure and the associated optimisation problem, while Section 4 develops the algorithmic design. In Section 5 we report on the results and discuss them. Section 6 concludes the work, outlining limitations and future directions.

## Literature review

**Fig. 1:**
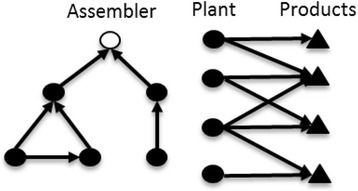
An assembler’s procurement network and the associated plant-product bipartite network

The design of a product-plant network is concerned with the distribution of production responsibilities across a network of factories (Fig. 1). The design plays an important role in determining the cost and reliability of the resulting production output. The network can consist of external suppliers or production plants that are internal to the company (both terms used interchangeably hereon). At the design stage of such a network, several decisions need to be given, including, which plant will produce and deliver which product, at what quantity and frequency, given associated costs, and estimated service levels. The problem of creating designs has been addressed in the vast field of “Supply Chain Design” from various interlinked perspectives such as flexibility (Stevenson and Spring 2007), demand uncertainty (e.g. Tsiakis et al. 2001), supply uncertainty (e.g. Goh et al. 2007, Lin and Wang 2011), and also by various methodologies including analytical and simulation based modelling, as well as strategic planning.

Reliability has been linked to supply uncertainty (Qi et al. 2007, Lundin 2012) as a challenge and several diverse modelling approaches have been put forward as potential solutions (see Snyder et al. 2006 and Snyder et al. 2016 for comprehensive reviews) (Table 1).

**Table 1:** Example approaches to reliable supply chain design optimisation

Approach	Focus	Example references
*Sourcing flexibility*	Optimal number of suppliers under disruptions (routine sourcing) or use backup suppliers after disruption (contingent rerouting)	Berger et al. (2004a, b), Berger and Zeng (2006), Dada et al. (2007), Meena et al. (2011), Yu et al. (2009), Masih-Tehrani (2011)
*Facility location*	Optimal placement of facilities under uncertain demand/supply conditions	Hakimi (1965), Bundschuh et al. (2003), Church et al. (2004), Santoso et al. (2005), Snyder et al. (2006), Church and Scappara (2007), Garg and Smith (2008), Yu et al. (2009), Lim et al. (2010), Azad et al. (2013), Baghalian et al. (2013), Shishebori et al. (2014), O’Hanley and Church (2011)
*Vector assignment*	Distribute customer demand across multiple facilities based on distance based cost and demand/supply uncertainty	Weaver and Church (1985), Pirkul (1989)
*Maximum coverage*	Maximise coverage of demand served from a facility under demand uncertainty and congestion	Church and Re Velle (1974), Daskin (1988)
*Process flexibility*	Optimal placement of redundant processes and capacity to meet uncertain demand	Jordan and Graves (1995), Barad and Nof (1997), Graves and Tomlin (2003), Barad (2003)
*Network reliability*	Connectivity of nodes under disruptions	Colbourn (1987), Shier (1991), Shooman (2002), Barrera et al. (2015)

*Sourcing flexibility* is concerned with the costs and trade-offs associated with buying from multiple suppliers (Berger et al. 2004a, b), determining the optimal number of suppliers (Berger et al. 2004a, b Berger and Zeng 2006), determining trade-offs when suppliers do not offer perfectly substitutable goods (Dada et al. 2007), and smoothing out disruptions different facilities are subjected to different demand or disruption patterns (Yu et al. 2009). This literature is mostly from a dyadic perspective in that a focal firm and not a network of firms. Furthermore, very few studies have investigated the assembly of products and instead focus has been on a single product distributed across several suppliers. Masih-Tehrani (2011) have argued that in a network where products are distributed over multiple suppliers risk diversification is preferred, whereas in a system where a focal company assembles multiple products, risk concentration is preferred. While this deduction is an important lesson, in a real–life supply system, a supplier could produce multiple products, and a product could be produced by multiple suppliers (Brintrup et al. 2015). There is a lack of models that can find trade-offs between cost and reliability while addressing both of these aspects.

*Facility Location* approaches are concerned with where should inventory be stored and distributed from, but does not take into account multiple products distributed across the supply network. Reliability research in Facility location problems investigates where and how much to place under demand uncertainty (e.g. Yu et al. 2009), more recently, supply uncertainty (Snyder 2006, Baghalian et al. 2013). For example, Bundschuh et al. (2003) and Azad et al. (2013) propose network robustness optimisation models, which determine both facility locations and assign customers to facilities. Shishebori et al. (2014) optimise facility location with a constraint on maximum allowable disruption cost. Church et al. (2004) propose a model to identify the best location of facilities in the case of a maximally disruptive failure. In their review, Snyder et al. (2006) and Synder et al. (2016), note that considerably fewer papers discuss disruptions within the context of supply network design, which our work contributes to. Within this strand, Santoso et al. (2005) has produced a network model for demand uncertainty, and Garg and Smith (2008) determine the minimum set of links to be constructed for a given set of failures.

Reliable facility location researchers have distinguished between design of facilities and fortification of facilities after a disruption occurs (Snyder et al. 2006). The key difference between these models is that in the former facilities can be placed anywhere, whereas in the latter facility locations are fixed, and the maximum damage is minimised through by choosing facilities for increased fortification, such as inventory placement (Hakimi et al. 1965, Church and Scappara 2007, O’Hanley and Church 2011, Lim et al. 2010). Our work investigates the design of a network where some proxy or historical information on supply reliability is available. Previous work has also distinguished between disruptions that results from events exogenous to the network and endogenous disruptions (Snyder et al. 2006). The work presented in this paper focusses on disruptions that occur endogenously, and are independent from one another.

In *Vector Assignment* models, each customer is assigned to multiple facilities using a demand frequency based on the customer’s distance from the facility (Weaver and Church 1985, Pirkul 1989), and based on the reliability of suppliers. In a similar vein to Vector assignment, *Maximum Coverage* models maximise demand served from a facility and are used for example for designing emergency services given probabilities of demand (Church and Re Velle 1974), and congestion on the network (Daskin et al. 1988). Both approaches do not consider multiple product types and are at the dyadic level investigating a path length of one between demand and supply.

Other studies have attempted formulating supply reliability as an allocation problem under the headings of *Process Flexibility*. Jordan and Graves (1995) put forward the “chain” concept, which they define as a bipartite network of products and plants. However, they model demand uncertainty, rather than supply, and model a chain where there is redundant capacity in plants for producing alternative products as a response to demand fluctuations. They model the excess capacity and cost trade off, proving that a small amount of redundant capacity can have the benefits of total flexibility. Graves and Tomlin (2003) then extend this work to supply chains that produce multiple products and have consistent findings. Jordan and Graves (1995)‘s work resulted in various analytical models. Many researchers created bottom up models, where uncertainty is modelled alongside inventory production flow parameters, and then addressed by redundant processes (Barad and Nof, 1997), capacity planning (Jordan and Graves 1995), or logistics channels (Barad 2003). In their review Stevenson and Spring (2007) argued that while these models provide useful insights, they have limited relevance to the more complex network structures found in practice.

Stevenson and Spring (2007)‘s point is supported by empirical samples of real life supply chains show complex network structures rather than “chains” or “echelons”. For example, studies on the global automotive network showed that there is a 21% chance of 2 first tiers sharing links with one another (Brintrup et al. 2016). Lomi and Pattison (2006) found triadic motifs in the Fiat Panda network. While empirical studies display complex network structures, corresponding modelling activity in supply chain design has been left behind.

The field of network science might provide us with some answers. In this field, assessing a complex network’s reliability is usually formulated as a vulnerability problem. Studies extract the underlying problem domain as a graph G (N, L) with a set of Nodes N and Links L between the nodes and study topological connectivity after potential disruption events. As many real life networks have heterogeneous distribution of connectivity, removal of certain nodes or links would result in greater damage to the network than others. Global measures that assess vulnerability include operational pairs (Grubesic et al. 2008), operational paths (Jenelius et al. 2006), minimum shortest paths, cyclomatic number, maximum network circuits, alpha index, beta index (see Newman 2010 for a review). On the other hand, local measures examine the individual nodes or links whose damage would impact the network the most. These include betweenness centrality, degree centrality, closeness centrality, eigenvector centrality amongst others (Borgatti and Everett 2006, Ledwoch et al. 2017). Critical edge definition methods assess the minimum number of nodes and links whose removal would disconnect the network (e.g. Duque-Anton et al. 2000, Goyal and Caffery 2002, Jorgic et al. 2004). Dinh et al. (2010) argued that none of these measures are able to consolidate disruption scenarios at the global scale, and created a pseudo approximation algorithm to find the minimum set of nodes whose removal will result in a given amount of degradation (pairwise connectivity) to the network.

Research that examines the optimisation of a network’s reliability is concerned with maximising the probability that a network will remain connected after disruptions (Colbourn 1987, Shier 1991, Shooman 2002, Barrera et a 2015). However creation of network vulnerability (or conversely, reliability) measures for supply networks is not straightforward because of key differences between these models and the supply network application domain. First of these is that there is not only topological connectivity between plants; but also a distribution of products across those plants, which are procured to create a final assembly. In other words, the model needs to be from the point of view of a focal node that needs to assemble resources distributed over a given network. Furthermore, while network k models are concerned with connectivity, supply network models discuss costs that result from incorporating reliability and that result from a disruption (Synder 2006). Lastly, it is important to note that supply chains show dependencies where each path needs to be traversed to make the end product, which is different from the structure of, for example, communication networks where the links typically indicate possible alternative pathways.

Furthermore, most network reliability studies focus on connectivity and assume heterogeneous node or link contribution to reliability i.e. each node or edge that is removed from the network has the same characteristics. In real life, a supply network would include links that would differ in terms of both their reliability and cost i.e. delivering from London to Manchester would be more straightforward than delivering from London to Niger.

Based on the extant literature, our contribution is thus threefold: We bring the problem of reliable supply chain design and network science domains together by framing the supply chain configuration problem as a network design optimisation problem and developing a measure for a manufacturer’s reliable access to products across plants. This allows us to incorporate networks structures into the design of a more realistic product-plant configuration, and at the same time making it possible to model multiple suppliers producing the same product type, and suppliers producing multiple product types. Second, we formulate the problem as an optimisation problem by designing the necessary decision variables, objective functions and constraints. Finally, we analyse the use of genetic algorithms to solve the optimisation problem by applying it to an automotive producer’s plant-product network.

## Problem description and formulation

**Fig. 2:**
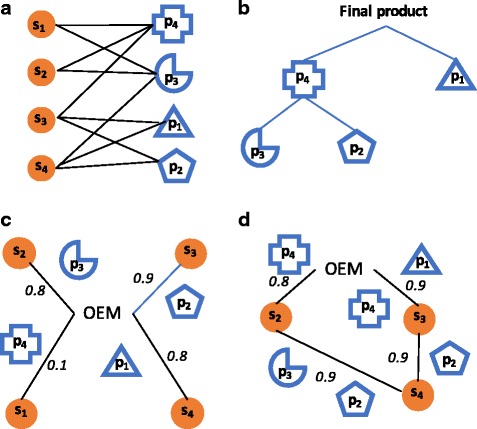
Illustrative supply chain reconfiguration (**a**) bipartite network of suppliers and products (**b**) final product structure (**c**) initial unreliable network configuration (**d**) more reliable network configuration

A graphical depiction of the reliable supply network design problem is illustrated in Fig. 2. Each of the four suppliers have multiple capabilities, and the cost of producing a product in different plants is variable. Links between each of the suppliers, and between the main assembler (OEM) and suppliers are both possible. Each potential link has a given reliability score and cost. Thus multiple supply chain configurations can be created for the OEM to access all products necessary for the assembly. The OEM may procure one unique product from each of the suppliers (Fig. 2a). However, Supplier 1 is not a reliable supplier as it has a low score (*0.1*) – if this supplier fails, the assembly cannot be made. An alternative configuration might include the OEM procuring directly from Suppliers 2 and 3, both of which procure from Supplier 4. In this configuration Product 4 is multi-sourced to two suppliers in order to incorporate redundancy in the design. Suppliers are assigned multiple products. This design seems to be more reliable, but is it the most reliable and cost effective? A systematic method that considers trade-offs in both the cost of the configuration, and the reliability of alternative configurations needs to be developed. In this section, we first create a measure that evaluates the reliability of the design (Section 3.1) and next formulate an optimisation problem that systematically balances costs against reliability (Section 3.2).

### Assessing reliability

Consider that an assembler needs to procure a set of Products $\overline{P}$ from a set of Production plants $\overline{Q}$to create a final consumer facing product. The connections between production plants can be denoted by the binary matrix ***L,*** where

$$ {L}_{ij}:= \left\{\begin{array}{c}1,\mathrm{if}\  \mathrm{Plant}\ i\ \mathrm{sends}\  \mathrm{products}\  \mathrm{to}\  \mathrm{Plant}\ j\ \\ {}0,\mathrm{otherwise}\kern11.75em \end{array}\right.,\kern1em i,j=1..m, $$

and *m* is the number of plants. Further, the products provided by each Plant can be denoted by matrix ***F***, where:

$$ {F}_{ij}:= \left\{\begin{array}{l}1,\mathrm{if}\kern0.17em \mathrm{Plant}\;j\;\mathrm{produces}\kern0.17em \mathrm{Product}\;i\\ {}0,\mathrm{otherwise}\end{array}\right.,i=1..n,j=1..m, $$

and *n* is the number of products.

If an assembler can access to a plant through a set of links, then that manufacturer has a “path” to the products of that plant. The products in facilities that supply directly to the assembler have a path length of 1 to the assembler. These plants might procure products from other plants, creating path lengths of 2; who have a direct link to a plant of path length 1, but no direct link to the assembler. Thus, let us call the number of links the assembler needs to traverse to access a certain product, path length *r*.

The total number of paths of length *r*, ***N***^(***r***)^, in the network ***L*** between any two vertices *i* and *j* is given by (Newman 2010):

$$ {N}_{ij}^{(r)}={\left[{L}^r\right]}_{ij}, $$

where ***L***^***r***^is *r*^th^ power of matrix ***L***.

Thus by extension it can be shown that the number of paths of length *r* between node *j* and product *k* on the network can be calculated as:

$$ {A}_{kj}^{(r)}=\sum \limits_{i=1}^m{F}_{ki}\cdot {\left[{L}^r\right]}_{ij}. $$

The above representation thus can include a redundancy of products and redundancy of paths to products. In other words, the resulting configuration can include duplicates of products, and more than one path to the said product.

An adjusted matrix $\overline{\boldsymbol{L}}$can be created when the reliability of links in ***L*** can be estimated a priori as a constant value in the interval [0,1], with 1 being the most reliable and 0 the least reliable,

$$ {\overline{L}}_{ij}={L}_{ij}\cdot \mathit{\operatorname{Re}}l\left({L}_{ij}\right), $$

where *Rel*(*L*_*ij*_) is the reliability score of the link between *i* and *j*.

The adjusted $\overline{\boldsymbol{A}}$ becomes:

$$ {\overline{A}}_{kj}^{(r)}=\sum \limits_{i=1}^m{F}_{ki}\bullet {\left[{\overline{L}}^r\right]}_{ij}. $$

Here, each column sum would denote the reliability adjusted access level of each plant to all products in the network. Since we are interested in the assembler’s access to all products, the first column of $\overline{\boldsymbol{A}}$ is the assembler’s reliability-adjusted path to each product in the network. Summing over this column would thus measure the manufacturer’s overall reliability in accessing to all the products in the network. This is defined as:

$$ \alpha =\sum \limits_{k=1}^n\sum \limits_{r\in \overline{r}}{w}_r\bullet {\overline{A}}_{k1}^{(r)}, $$

where $\overline{\mathrm{r}}$ is the set of path lengths that the analyst wishes to consider. Each path length r can be adjusted with a corresponding set of weight w_r_. For example we may choose to consider the reach to products from path lengths of 1 to 2, with a decrease in the significance of path length with weights 1 to 0.5.

Note that the assembler is also represented in ***F*** and the first row of ***F*** is 0 as the assembler does not produce products but only procures them. In the case that the assembler also produces products, we represent the assembler by a supplying plant, and add a dummy assembler in row 0 with a single link to the actual assembler whose reliability is 1.

### Balancing reliability and costs

Suppose now that each link of ***L*** incurs a cost of procurement, denoted by a matrix ***M***. Then.

$$ \boldsymbol{N}=\boldsymbol{M}\ast \boldsymbol{L}, $$

where operation * denotes element-by-element multiplication of matrices, gives the cost of each link on the specific network configuration. Further, the cost of producing a product in a given plant in $\overline{Q}$ can be denoted by matrix ***K***. The production cost is an aggregate value that the decision maker can model based on manufacturing variables such as labour, raw material and operational costs, and holding costs. Then

$$ \boldsymbol{G}=\boldsymbol{K}\ast \boldsymbol{F} $$

gives the cost of production on the specific network configuration. The total cost of a given configuration becomes.

$$ \boldsymbol{C}=\boldsymbol{N}+\boldsymbol{G}. $$

The problem of finding the maximally reliable network configuration ***L*** with minimal cost can be laid out as a bi-objective optimisation problem:

$$ \alpha =\sum \limits_{k=1}^n\sum \limits_{r=1}^{\overline{r}}{w}_r\bullet {\overline{A}}_{k1}^{(r)}=\sum \limits_{k=1}^n\sum \limits_{r=1}^{\overline{r}}{w}_r\bullet \sum \limits_{i=1}^m\ {F}_{ki}\bullet {\left[{\overline{L}}^r\right]}_{i1}\to \underset{L}{\max }, $$

$$ \boldsymbol{C}=\boldsymbol{N}+\boldsymbol{G}=\boldsymbol{M}\ast \boldsymbol{L}+\boldsymbol{K}\ast \boldsymbol{F}\to \underset{L}{\min }. $$

Three constraints need to be designed. First of these is that plants cannot supply to themselves. Hence the diagonal of the ***L*** must be 0:

$$ {L}_{ii}=0,\kern1em \forall i=1..m. $$

Secondly, the network must be a connected network i.e. every plant must have at least one link to either another plant or the assembler.

Two options exist for imposing the connectivity constraint. First of these is solving analytically using the algebraic connectivity rule, which states that network is connected if the second smallest eigenvalue of the Laplacian matrix of ***L*** is positive (Fiedler 1973). However, for this solution to be viable, ***L*** needs captured in a symmetric and unidirectional form. The second option is the deployment of network search algorithms for each solution found. Examples include Dijkstra, Floyd-Marshall, Kosaraju, or simple depth-first or breadth-first search algorithms. If the network is connected; every node will be accessed within a finite amount of time. However, deploying the search method would be computationally expensive as each iteration of the optimisation algorithm would re-trigger the search. As the network size grows, search algorithms will be increasingly unscalable, especially with heuristic methods that generate multiple solution instances at each iteration. We therefore opt for the analytical option.

We first capture ***L*** in a symmetrical undirectional form $\tilde{L}$:

$$ {\tilde{L}}_{ij}:= \Big\{{}_{0,\mathrm{otherwise}}^{1, if\;{L}_{ij}=1\; or\;{L}_{ji}=1},i,j=1..m. $$

Then the degree matrix ***D*** of $\tilde{\boldsymbol{L}}$ is created. The degree matrix is a diagonal matrix which contains information about the degree of each node, where the degree of a node is the number of links attached to each node:

$$ {D}_{ij}:=\left\{\begin{array}{c}\deg \left(\tilde{l_i}\right),\mathrm{if}\ i=j\\ {}0,\kern0.5em \mathrm{otherwise}\end{array}\right., $$

where

$$ \deg \left(\tilde{l_i}\right)=\sum \limits_{j=1}^m{\tilde{L}}_{ij} $$

is the degree of node *i* in network $\tilde{\boldsymbol{L}}$.

The Laplacian matrix ***L***^′^ is obtained by subtracting $\tilde{\boldsymbol{L}}$ from ***D***:

$$ {\boldsymbol{L}}^{\prime }=\boldsymbol{D}-\tilde{\boldsymbol{L}}. $$

The second constraint thus is:

$$ {\lambda}_2\left({\boldsymbol{L}}^{\prime}\right)>0, $$

where *λ*_2_(∙) is the second smallest eigenvalue.

The final constraint is that every supplier must produce at least one product:

$$ \sum \limits_{i=1}^n{F}_{ij}\ge 1\kern0.75em \forall j=1..m. $$

To summarise, the optimisation problem is:

$$ \alpha =\sum \limits_{k=1}^n\sum \limits_{r=1}^{\overline{r}}{w}_r\bullet \sum \limits_{i=1}^m\ {F}_{ki}\bullet {\left[{\overline{L}}^r\right]}_{i1}\to \underset{L}{\max }, $$

$$ \boldsymbol{C}=\boldsymbol{N}+\boldsymbol{G}=\boldsymbol{M}\ast \boldsymbol{L}+\boldsymbol{K}\ast \boldsymbol{F}\to \underset{L}{\min }. $$

Subject to:

$$ {L}_{ii}=0,\kern0.75em \forall i=1..m, $$

$$ {\lambda}_2\left(\boldsymbol{D}-\tilde{\boldsymbol{L}}\right)>0, $$

$$ \sum \limits_{i=1}^n{F}_{ij}\ge 1\kern0.75em \forall j=1..m. $$

## Algorithm design

The formulation presented in the previous section constitutes a multi-objective network optimisation problem, which is not well handled in network literature (Newman 2010). Heuristic methods have been promising. Of these genetic algorithms (GA) has been a common tool for optimising networks (Nurika et al. 2014). Some of the most relevant works include that of Ahn and Ramakrishna (2002) who apply genetic algorithms to shortest path finding in a routing problem and study the effect of population size. Ishrat and Ali (2013) created a genetic algorithm approach to finding feasible paths in a dynamic mobile adhoc network routing problem. Genetic algorithms have been popular in routing, load balancing and bandwidth assignment problems in wireless networking (Mehbood et al. 2014), as well as supply chain design and configuration problems (e.g. Altipar mak et al. 2006, Farahani and Elahipanah 2008, and more recently, Lee et al. 2015 and Yuce et al. 2014). A review of workability of genetic algorithms in optimising networks is given by Nurika et al. (2014).

We opt for the use of GA as this class of algorithms are reported to handle unconventional search spaces well. Additionally, at each iteration the GA returns a population of solutions rather than an individual, which would help reduce the computational cost of the problem. Furthermore, the bit string representation used in GA can be leveraged for the binary matrices **L** and **F** inherent to the problem.

**Fig. 3:**
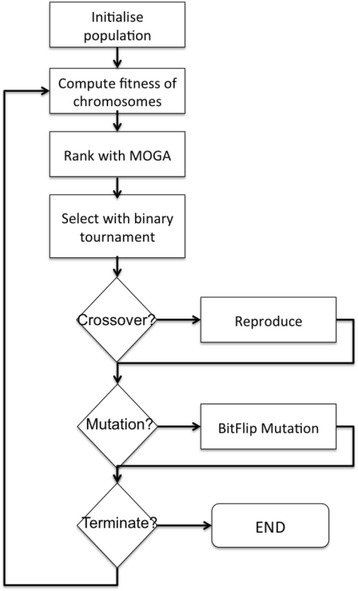
Algorithm flowchart

The GA formulation of the problem includes 5 parts (Fig. 3):

1. *Chromosome:* GA operate by defining an initial population of candidate solutions each of which is called an individual. Individuals are encoded as a digital “chromosome” that represents the variables of the optimisation problem to be solved. Each bit is called a gene. Several genetic encoding styles are possible. In our case we opt for binary string representation as this would handle the adjacency matrix and the binary product-plant matrix naturally. The first part of the bit string is used for L whereas the last part is used for the **F**. As **L** is symmetric and has a diagonal of 0, only n(n-1) bits are used, where n is the number of plants. As **F** the first row of **F** is 0, m(n-1) bits are used where m is the number of products.
2. *Fitness function*: The fitness function is essentially the objective function or functions used to assess the “fitness” of the chromosomes. These in our case are *α and* ***C.*** Fitter chromosomes have a higher probability to pass their genes onto the next generation.
3. *Selection*: Selection is the mechanism by which fitter chromosomes are given a bias to pass on their genes to the next generation. We use the Binary Tournament selection operator. Because we have two objective functions, our problem constitutes a multi-objective optimisation problem. In multi-objective optimisation there is no single optimum solution, but a set of *Pareto optimal* solutions. Pareto optimal solutions are trade-offs between different objectives and are also called non-dominated solutions, meaning that there is no other solution which would improve an objective without causing a worsening in at least one of the other objectives (Deb 2001). Given the nature of our problem, non-dominated sorting algorithms are needed to rank chromosomes according to their fitness in both objectives. For this we experiment with three popular multi-objective optimisation algorithms NSGA2 (Deb et al. 2002), SPEA2 (Zitzler et al. 2002), and PAES (Knowles and Corne 1999) implemented in the jMetal framework (Durillo and Nebro 2011).
4. *Reproduction:* Once fit chromosomes are selected for reproduction, the crossover process is initiated to exploit their best traits by mixing them to improve their fitness. At each crossover two parents are selected to reproduce two offspring chromosomes. We use the Heuristic Uniform Crossover (HUX) over half the bits that differ between the two parent chromosomes. This is done by first finding the bit positions that differ between the two parents and then randomly selecting a differing position to swap the bits. The process is repeated until half of the differing bits have been swapped.
5. *Mutation*: The mutation operator produces random changes in the chromosome. The operator is used to explore previously unexplored search space and helps diversify the population, preventing premature convergence. We use the BitFlip mutation, which takes the chosen chromosome and inverts the bits. In BitFlip mutation rather than selecting a single bit to mutate, the operator finds two random points in the string and reverses the order of the bits between those points.

**Fig. 4:**
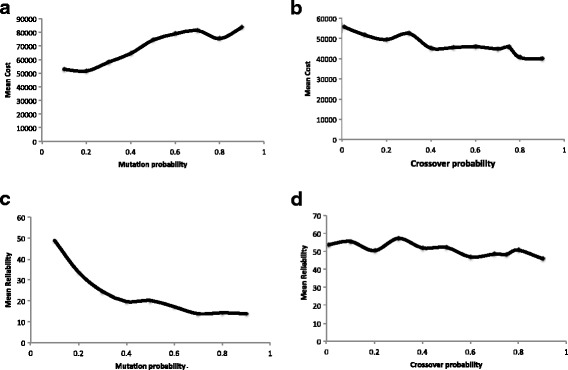
Performance of NSGA2 with different crossover and mutation rates (on Problem 1). (**a**) Mean cost as a function of mutation probability. (**b**) Mean cost as a function of crossover probability. (**c**) Mean reliability as a function of mutation probability. (**d**) Mean reliability as a function of crossover probability

Experiments were carried out on an Armari Magnetar, 3.5 GHz, 64 GB RAM Workstation. Computational trials indicated that setting the total number of generations N_max_ to 25,000 and the population size to 100 achieved a good balance between solution quality and computational efficiency. Figure 4 shows the solution quality of NSGA2 for five selected instances with different value of probability of crossover ranging from 0.1 to 0.9 (with an increment of 0.1). It appears that a crossover rate of 0.9 and a mutation rate of 0.1 leads to better solution quality. Thus in our computational experiments, the probability of crossover and mutation were fixed to be 0.9 and 0.1 respectively.

## Results

**Fig. 5:**
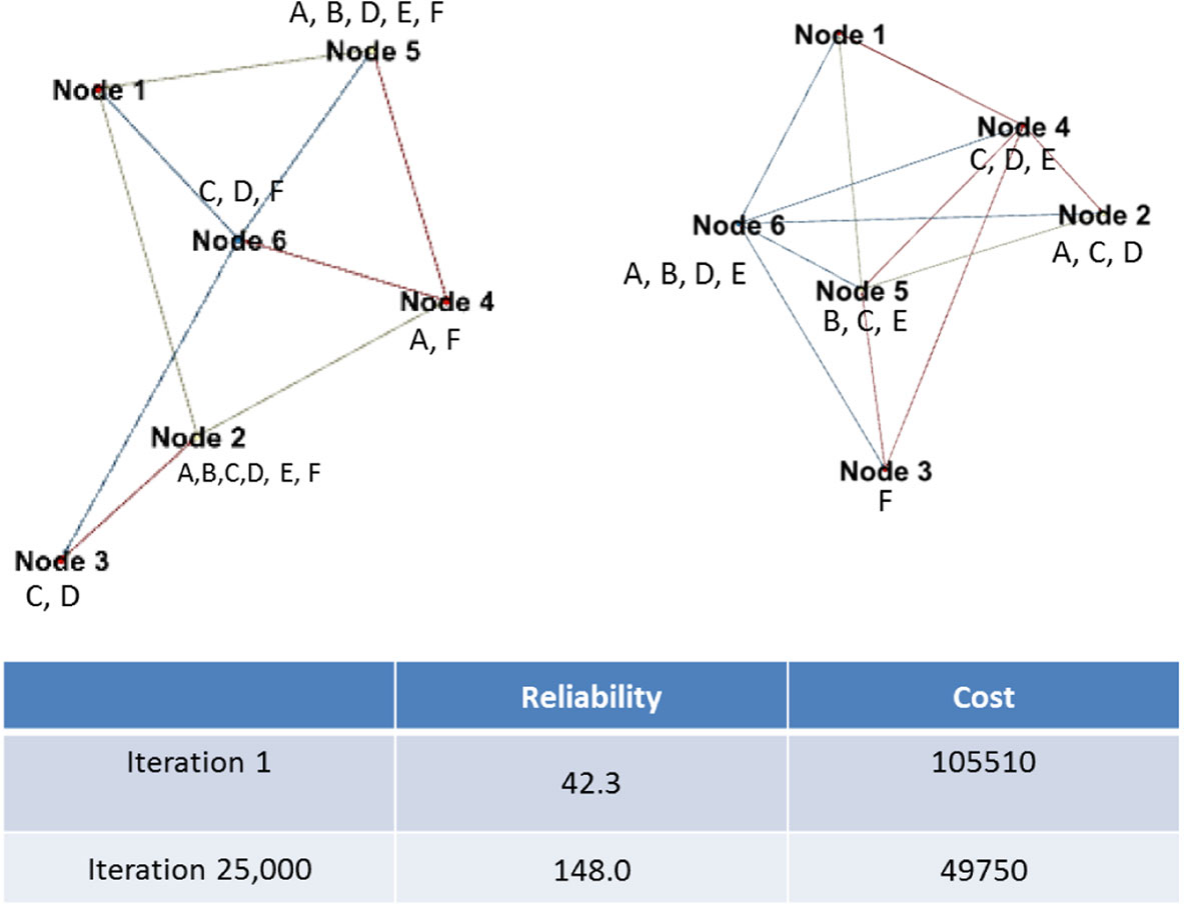
Example evolution of network configuration

The approach is tested with an automotive light and lamp producer’s production network (Table 2, Problem 1). The network consists of one assembler, 5 plants (Nodes 1–5) and 5 product categories (Products A-F) distributed over these plants. Figure 5 shows a randomly selected member of the initial population with an average reliability score of 42.3 and an average configuration cost of 105,510; and a member of the Pareto front obtained with NSGA2 with a reliability score of 113 and cost of 43,750. Figure 5 illustrates the search process of NSGA2 on this problem instance. The evolution process indicates initial solutions are improved effectively.

**Table 2:** Multi-objective optimisation performance metrics on three problem instances

Algorithm	Problem instance	Solution space	Number of nodes	CPU (ms)	HV	GD	Spread
NSGA2	1	3025	6	63,221.8	0.824	0.003	0.771
	2	29,241	10	65,144.2	0.855	0.006	0.670
	3	164,836	15	72,203.8	0.699	0.017	0.878
SPEA2	1	3025	6	67,348.8	0.800	0.004	0.841
	2	29,241	10	69,770.6	0.548	0.008	0.720
	3	164,836	15	74,207.0	0.642	0.029	0.820
PAES	1	3025	6	59,784.6	0.433	0.084	1.560
	2	29,241	10	73,017.0	0.146	0.026	1.716
	3	164,836	15	71,253.8	0.399	0.130	1.481

Each experiment has been run 30 times. GA parameters are kept same across

In this case, the production responsibilities of the unreliable nodes 2 and 3 are reduced, and the more reliable Nodes 4 and 6’s responsibilities are increased. Node 1, which is the assembler node, preserves its incoming link degree of 3; however the total number of links in the network has increased from 10 to 12. The overall product distribution is more even than the initial solution, which is facilitated by additional linkages between plants.

**Fig. 6:**
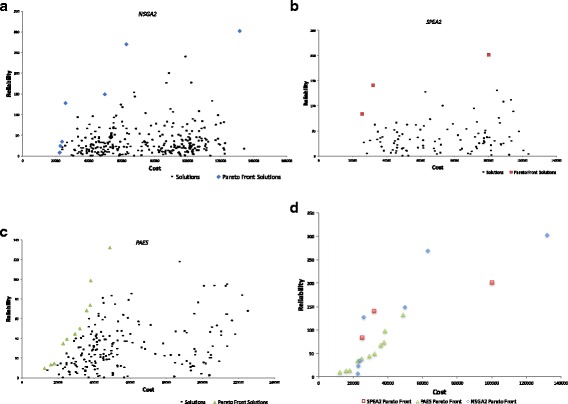
Performance of (**a**) NSGA2, (**b**) SPEA2, (**c**) PAES, and (**d**) Comparisons of Pareto fronts generated by each algorithm (Problem 1)

Figure 6 shows the comparative performances of each multi-objective algorithm used in the optimisation process and the respective Pareto fronts obtained. Whilst the PAES seem to capture only a narrow range of the search space, NSGA2 has obtained the best spread across the non-dominated front, dominating solutions obtained from both the SPEA2 and PAES. The PAES has found a range of lower cost solutions the NSGA2 has found significantly more reliable configurations at slightly higher costs.

**Fig. 7:**
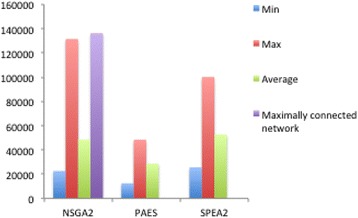
Comparison of Minimum, Maximum and Average Costs obtained by each algorithm to a fully connected network

The average, maximum and minimum cost of Pareto front solutions obtained by each algorithm on this problem is given in Fig. 7. Note that the method does not necessarily lead to highly connected networks and instead searches for trade-offs between cost and reliability, by for example deleting links from an unreliable supplier and assigning its products to a more reliable supplier. This is evident on the Pareto front obtained by the algorithms in Fig. 6 as the algorithm does not result in the maximal cost, which would have been obtained from a fully connected network. NSGA2 offers the most diverse set of solutions approaching both maximally and minimally connected networks as well as a range of solutions in between, showcasing the advantage of utilising this algorithm.

Further tests were conducted on progressively larger problem instances (Table 2). While Problem 1 is a real-life case study, Problems 2 and 3 are randomly generated problems using Problem 1 as a benchmark. The results are compared using three multi-objective optimisation performance metrics (Coello 2007). The use of these metrics is further motivated by recent applications in the field of supply chain design (e.g. Moncayo-Martinez et al. 2016):

- Hypervolume (HV): This metric represents the fraction of the objective space that is dominated by the obtained solutions, indicating the coverage of the real Pareto front. The larger the hypervolume, the better the optimiser (Coello et al. 2007).
- Generational distance (GD): This metric calculates the proximity of the solutions obtained to the population of the real Pareto front. The smaller the GD, the better the optimisation result (Veldhuizen and Lamont 1998).
- Spread (S): This indicator estimates the diversity of the set of solutions obtained on the Pareto front (Deb 2001). A smaller value of Spread indicates a more diverse set of non-dominated solutions.

We also report the CPU time for finding best solutions. Due to the probabilistic nature of GA, algorithms were run 30 times for each problem instance. Expectedly, as problem size increases so does CPU time, GD, and S, while HV decreases. PAES performs consistently worse than NSGA2 and SPEA2 in all metrics, while NSGA2 outperforms SPEA2 in HV and CPU time, albeit slightly. It appears that NSGA2 maintains its performance across the three problem instances, dealing with the product-plant configuration problem better than the other two algorithms. The different performances of these algorithms highlight the fact that the optimisation algorithm choice has an important role in achieving a good trade off solutions in the reliable supply network design problem formulated in this paper.

## Conclusions

### Summary and key results

Although supply chain design literature offers many bottom up models for plant-product configuration, the reliability of resulting designs have received little attention. Bottom up models require detailed models of material flow, and thus are complex to build and solve, and difficult to generalise beyond the case they are designed for. Furthermore, supply chain designs typically focus on chain like structures and do not take network formations into account, despite a growing number of empirical work suggesting that real life supply chains contain network structures.

In this paper we developed a top down model for evaluating the reliability of production over a network of plants in a given supply chain configuration. We assumed that the supply chain is reconfigurable, hence the problem may be viewed as a product- plant network configuration problem. The model is generic as it works with minimum information, and can handle network structures. We then used the model as a basis to frame the configuration problem as a multi- objective optimisation problem that balances cost against reliability. We opted for the use of genetic algorithms because of their ability to handle unknown search spaces at reasonable computational speed. Of the three algorithms NSGA2 has achieved the best results in terms of Pareto front spread. Algorithms differed considerably in their performance, meaning that the choice of algorithm has significant impact in the resulting search space exploration.

### Limitations and future research opportunities

Several assumptions underlie the problem formulation presented in this work; each of which present an opportunity for further improvement to the model.

Firstly, we assume that the reliability of links between suppliers can be a priori estimated and that this estimation is accurate. Thus the application of the developed model would benefit from a structured decision process that enables a manufacturer to carry out such estimations such as the incorporation of historical data analysis.

Secondly, perfectly substitutable goods are assumed, although in real life supplier offerings may differ in their quality and therefore products may not be perfectly substitutable. Remedying this assumption could be done with additional costs to represent the substitutability of the products.

In addition, it is assumed that the reliability of a supplier is the same for each product it offers although different production constraints may result in different reliability scores for each product. In this case the model could be further developed with multiple links to products at each supplier.

The assembler is assumed to have no cost for accessing to alternative sources of products although buying from an alternative supplier or production plant could mean additional transaction costs. These could be easily incorporated into the model.

As our aim has been to create a generic model we deliberately ignored bottom up details such as material quantities and associated cost models. These could be built in as extensions to the model specific to the case study problem being addressed. Finally the formulation does not allow precedence constraints; meaning that the sequence with which resources must arrive at the assembler is not incorporated in the model.

Although the model has been designed with product-plant network configuration in mind, it also fills a gap in network science literature where a focal node’s ability to access resources distributed over a network is assessed.

## Nomenclature

$\overline{P}=\left\{1,2,\dots, n\right\}$ – set of products.

$\overline{Q}=\left\{1,2,\dots, m\right\}$ – set of production plants.

***L*** ∈ *R*^*m* × *m*^ – binary matrix representing connections between production plants.

***F*** ∈ *R*^*n* × *m*^ – binary matrix that describes what products are produced by each plant.

${N}_{ij}^{(r)}$ – number of paths of length *r* between plants *i* and *j* in the network ***L.***

${A}_{kj}^{(r)}$ – number of paths of length *r* between plant *j* and product *k*

*Rel*(*L*_*ij*_) – reliability score of the link between plants *i* and *j* in the network ***L***

$\overline{\boldsymbol{L}}$ – adjustment of matrix ***L*** that takes into consideration the reliability of links

*α* – objective function representing the reliability of the manufacturer in accessing all products in the network

w_r_ – weight in the objective function *α* that corresponds to the path of length *r*

***M*** – matrix representing the cost of procurement for each link between any two plants

***N*** – matrix that describes the cost of each link in the network ***L***

* – element-by-element multiplication

***K*** – matrix representing the cost of producing each product in each plant

***G*** – matrix that describes the cost of production in the network ***L***

***C*** – objective function corresponding to the total cost of the network configuration ***L***

$\tilde{\boldsymbol{L}}$ – representation of the network ***L*** in a symmetrical unidirectional form

$\deg \left(\tilde{l_i}\right)$ – number of links attached to node *i* (degree of node) in the network $\tilde{\boldsymbol{L}}$

***D*** – matrix describing the degree of each node in the network $\tilde{\boldsymbol{L}}$

*λ*_2_(·) – second smallest eigenvalue of a matrix
